# Development of the peer-supported open dialogue attitude and competence inventory for practitioners: A Delphi study

**DOI:** 10.3389/fpsyg.2023.1059103

**Published:** 2023-03-30

**Authors:** Vladimirs Fedosejevs, Jinyu Shi, Mark Steven Hopfenbeck

**Affiliations:** ^1^Division of Psychology and Language Sciences, University College London, London, United Kingdom; ^2^Essex Community Sentence Treatment Requirements Service, St Andrew’s Healthcare – Community Partnerships, Essex, United Kingdom; ^3^Department of Health Sciences, Norwegian University of Science and Technology, Gjøvik, Norway

**Keywords:** open dialogue approach, peer-supported open dialogue, Delphi method, inventory, PODACI, interviews, questionnaires

## Abstract

**Introduction:**

Peer-supported Open Dialogue (POD) is a novel approach to mental health care that is currently being practiced and researched in the United Kingdom. For POD to be successfully implemented, effective training must be provided to make sure trainees are prepared to deliver the approach as intended. Therefore, a specific instrument that can assess the development and competence of POD trainees, as well as the effectiveness of POD training is crucial. Therefore, the current study aimed to establish an inventory named the *Peer-supported Open Dialogue Attitude and Competence Inventory* (PODACI), measuring the changes in attributes and attitudes of trainees before and after training.

**Methods and Results:**

To generate the inventory, a four-round modified Delphi approach was used. We first identified the dimensions that are essential and specific to POD through an extensive literature review and individual interviews with practitioners (*n* = 8). After generating the items, we further refined the items through two rounds of questionnaires, asking practitioners to rate the relevance of each item from 1 (not essential) to 4 (highly essential; *n* = 21 and *n* = 10), and finalized the inventory *via* a focus group interview with POD trainers (*n* = 4). In total, 76 items were included in the PODACI. A good consensus on the items was reached: the median score of the items was all above 3.00 (essential) and achieved an agreement level greater than 85%. The Kendall coordination coefficient W was 0.36 and 0.28 in the two questionnaires employed, indicating a fair level of agreement between participants.

**Discussion:**

The PODACI provides a way to measure attitudinal and competency factors related to the treatment integrity of POD as well as the efficacy of the training courses being offered. This highly enriched instrument opens up a wide range of possibilities for POD research and application, facilitating the development of Open Dialogue services. The next step is to assess the psychometric properties of the inventory.

## 1. Introduction

Open Dialogue (OD) is a novel approach to mental health care that embodies systematic family therapy, delivering a distinct form of therapeutic dialogue (Seikkula et al., 2006). For OD, the main aim of the clinicians is the creation of a common understanding of a presented difficulty through shared language, rather than problem-solving. OD is based on the principle that both the clients and clinicians are people with their own experiences, and when they are able to work collaboratively, can help achieve an understanding of the situation. The engagement of every party in the treatment and the transparent nature of therapy planning is what the term *open* refers to Olson et al. (2014). To encourage free exchange and break down the clinician-‘patient’ boundary, OD focuses on *dialogue* both as a method of therapy and a system of care. For a network meeting (i.e., meeting with the client and their social network) to be dialogical, it needs to be based on the client’s own input rather than the agenda or specific targets of the clinicians. Therefore, clinicians need to have two essential skills to successfully practice dialogical therapy: the skill of responding and the ability to reflect. The former requires the clinician to pay attention to the utterances given by the client, the network members, and even themselves. The latter refers to the ability to reflect on the topics and the clinician’s own feelings that emerge in a meeting. These person-centered meetings facilitate listening, invite all voices to be heard, and construct meaning through seeing, hearing, and feeling all those present. It has been argued that only through a dialogical approach can one explore possible traumas that are often the root cause of severe symptoms of a mental health crisis (Olson et al., 2014). While OD incorporates principles of family therapy (i.e., adopt a network-wide exploration), it does not focus on the family system or the communicative patterns among the family (Seikkula, 2003) *per se*: OD does not aim to change the fixed dynamic of a system, but rather to create a joint space for new language, facilitating the production of different meanings for the particular difficulty (Seikkula, 2003). It is such features that differentiated OD from family therapy. Open Dialogue is also seen as a foundational framework for organizing and delivering help, involving the network, and creating a polyphony of voices at the point of initial contact with services, rather than an additional, time-limited intervention as is often the case with family therapy (Jackson and Thorley, 2021).

So far, there has been a growing body of supporting evidence for the application of OD. One of the first studies looking at OD’s effect on the treatment of first-episode psychosis came from Finland. Seikkula et al. (2006, 2011) reported that 70% of participants treated *via* the OD approach returned to their studies and work, with 82% showing no residual psychotic symptoms. Positive outcomes were still present even after 5 years, where the OD group (*n* = 42) had a smaller duration of untreated psychosis, reduced medication use, and fewer days in the hospital compared to the control group. The benefits of OD have also been consistently demonstrated in more recent studies across the world, including Finland (Granö et al., 2016; Bergström et al., 2018), United States (Gordon et al., 2016; Rosen and Stoklosa, 2016; Freeman et al., 2019; Gidugu et al., 2021), Denmark (Buus et al., 2019), and Australia (Dawson et al., 2021).

### 1.1. Open dialogue in the United Kingdom: Peer-supported open dialogue

Following the successful implementations of OD around the world, practitioners and researchers in the United Kingdom started to explore the practicality of a novel OD model: Peer-supported Open Dialogue (POD) (Razzaque and Stockmann, 2016). Besides OD’s fundamental principles (Seikkula et al., 2006; Olson et al., 2014), POD also involves peer workers with experiences of mental health services and are qualified to enhance the democratic nature of the POD meetings. Although the National Health Services (NHS) in the United Kingdom has a limited amount of POD services at the present, there is an actively growing interest in the approach. For instance, the ODDESSI (Open Dialogue: Development and Evaluation of a Social Network Intervention for Severe Mental Illness) is a large-scale program that is currently taking place in the country (runs from 2017–2022 but delayed due to COVID-19; Pilling et al., 2022). The program aims to assess the effectiveness, acceptability, and ability to implement POD into the NHS services. In line with the ODDESSI, several small-scale qualitative studies have revealed that POD allowed the clients to build a more equal relationship with their practitioners and made them feel listened to and acknowledged (Tribe et al., 2019; Hendy and Pearson, 2020; Twamley et al., 2021; Kinane et al., 2022).

Despite the positive evidence on POD, it is uncertain whether the approach can be implemented successfully in the NHS. Since the NHS is biomedically founded, emphasizing specific standards such as the risk management of each client (McKeown et al., 2015a,b) and medication as a possible solution of a ‘mental illness’ (Elliott et al., 2018), its focus differs from the core principles of POD. POD values a more unifying approach to mental health care, aiming to develop dialogical communication between the patient and their support system as a therapeutic intervention (Razzaque and Wood, 2015), and considering a wide range of factors and solutions that are primarily directed by the client. This difference is vital, as it changes the focus of the therapeutic meeting, but most importantly how people deliver mental health care and how future practitioners are trained. Indeed, identified by Razzaque and Wood (2015), POD practitioners themselves argued that implementing POD would be challenging due to (1) major cultural shifts from the medical-based treatment as usual (TAU) to a more person-centered, holistic, relational, and compassionate approach in POD (e.g., relying less on particular diagnosis, set procedures, and medical prescriptions, and putting more emphasis on collaborative decision making, hearing the voices of all present and creating a sense of safety so that all stories can be heard (Jackson and Thorley, 2021) (2) professional changes in current practitioners’ approach to mental health (e.g., surrendering one’s power and positive risk taking; Razzaque and Wood, 2015). While many clinicians embrace the possibility of creating a less oppressive medicalised service, challenging existing hierarchies within existing services is not easy (Tribe et al., 2019; Dawson et al., 2021). For individual practitioners, POD trainings can be difficult and somewhat uncomfortable as trainees are expected to work as part of a non-hierarchical team, share relevant aspects of their own life histories and display their emotional vulnerability (Schubert et al., 2021), which some of them described as almost a ‘cult-like culture’ (Florence et al., 2020). To narrow the cultural gap and help clinicians adapt to the changes, it is essential for them to receive effective and adequate training to practice POD efficiently.

### 1.2. Peer-supported open dialogue training

In POD, training plays a vital role in helping professionals to make necessary changes in their day-to-day practices, learn the key fundamentals of the approach, and deliver POD effectively, especially when they have been previously trained in different practices.

Currently, a one-year POD training course is being offered in the U.K. The training has now been running for almost 8 years (since October 2014) with hundreds of practitioners. The course consists of four residential weeks that are spread over the year and involves trainers from five different countries, including many of OD’s founders like Professor Jaakko Seikkula. To assess the efficiency of the training, Stockmann et al. (2019) conducted four focus group interviews with 27 trainees who completed the POD course. They found that the trainees reported the training as an emotional journey, which helped them to change their attitudes and approach to clinical work. In particular, POD training was considered to ‘re-humanise’ mental health practice compared to TAU, encouraging clinicians to be more authentic. The findings suggested that POD training promoted a different mindset that was almost inconceivable for participants who came from entirely different clinical backgrounds.

### 1.3. Treatment integrity in peer-supported open dialogue

One of the primary goals of professional training is to ensure treatment integrity. Treatment integrity is defined as “the degree to which treatment is delivered as intended” (Yeaton and Sechrest, 1981, 1992). Treatment integrity of an approach is also found to be positively correlated with the psychotherapy outcomes (Barber et al., 2007). Hence, any approach to mental health care should be able to be assessed with regards to the integrity of its implementation. Otherwise, the validity of treatment outcomes becomes limited, making it difficult to conclude the efficacy of the approach (Waltz et al., 1993).

Intervention integrity is often broken down into two overlapping but distinct areas: fidelity and adherence. The term fidelity is used to describe interventions at multiple levels including measures of systems implementation, service provision and operational principles, while adherence is used to describe the degree to which a practitioner delivers an intervention in accordance with theoretical and procedural elements of the model (Hogue et al., 1998). Adherence is closely related and often differentiated from the concept of therapist competence which can be defined as the internalization and integration of attitudes, knowledge, motives, beliefs, empathy, relational understanding, clinical reasoning, emotions, values, and critical self-reflection relevant to their practice (Epstein and Hundert, 2002; Baartman and de Bruijn, 2011; Perepletchikova, 2014; Cox et al., 2019; Crameri et al., 2020). In this sense, competence is what contributes to successful practice (Antera, 2021). Therapist competence captures important therapy process variables which have been shown to impact the therapist-patient alliance as well as treatment outcomes (Norcross and Wampold, 2011; Wampold, 2015).

Hence, any approach to mental health care should have the ability to assess a broad range of factors, including the competence and attitudes of the practitioners prior to using the approach. Otherwise, the validity of treatment outcomes becomes limited, making it difficult to conclude the efficacy of the approach (Waltz et al., 1993). In the ODDESSI trial, the fidelity of service delivery was measured using the COM-FIDE instrument (Alvarez Monjaras, 2019) and adherence was measured using the Open Dialogue Adherence Scale (Lotmore, 2019; Lotmore et al., 2023), but no instrument was included in the trial to measure competence.

Various mental health interventions have developed instruments to measure their practitioners’ or trainees’ competence. These instruments often took the form of scales that measure particular attributes (Grove et al., 2012), questionnaires that record knowledge and opinions, or inventories that are catalogues of different attributes, attitudes, and perceptions (Younas, 2017). For instance, researchers following the cognitive behavioral therapy (CBT) approach had developed multiple scales to measure treatment integrity, including but not limited to the Cognitive Therapy Scale (CTS; Vallis et al., 1986) and the 21-item Cognitive Therapy Adherence and Competence Scale (CTACS, Barber et al., 2003). A higher score on CTS was found to be associated with a greater decrease in the severity of clients’ depressive symptoms and anxiety after treatment, indicating that practitioners with greater treatment integrity delivered more effective treatment (Trepka et al., 2004; Strunk et al., 2010). The evidence suggested that instruments measuring treatment integrity offer a quantitative way to examine the effect of training and to decide whether the practitioners were readily trained, which is essential and beneficial for successful deliveries of the appropriate treatments.

Compared to interventions like CBT, POD is a newly emerged approach that needs more attention and research. OD is considered to be a ‘complex intervention’ due to the inclusion of several interacting components that are necessary for delivering a desired outcome (Lotmore, 2019). Before joining the POD course, every trainee has different starting points, experiences, and beliefs, so their journey throughout the training would vary individually. While some may find the training to be life-changing and are prepared to practice POD right away (Dawson et al., 2021), others may need more time to gain a better grasp of how to practice POD. Therefore, it is necessary to develop an instrument that assesses the development and competence of trainees before and after training (e.g., how well the trainee has internalized and integrated attitudes, knowledge, values, etc. relevant to their practice), further examining the integrity of POD delivery, as well as advancing our understanding of the efficacy of POD and facilitating its wider implementation.

### 1.4. Current study

The current study aimed to develop a self-report inventory called the *Peer-supported Open Dialogue Attitude and Competence Inventory* (PODACI). The inventory intended to examine (1) a trainee’s competence after training based on their attitudes and attributes and (2) the effectiveness of the POD training that is currently provided. To generate the inventory, we adapted a four-round modified Delphi procedure that combined a literature review, “expert” opinions, and group consensus through structured interviews and questionnaires (Joling et al., 2017; Keeney et al., 2017; Mao et al., 2020). The Delphi process has been shown to be highly effective in collecting data (Graefe and Armstrong, 2011), and well suited for areas with incomplete knowledge like POD (Skulmoski et al., 2007; Fink-Hafner et al., 2019). The procedure could strengthen the validity of the inventory with the inclusion of POD practitioners, trainers of the POD course, and current trainees. In this study, we first identified items that are specifically unique to POD through an extensive literature search and detailed discussion with POD practitioners, and then further refined the items through two rounds of questionnaires and one round of focus group interview.

## 2. Item generation

The first stage of the current study generated the initial sets of items through an extensive literature review as well as individual interviews with POD practitioners and trainers (i.e., Round One of the Delphi procedure).

### 2.1. Literature review

Before beginning the Delphi procedure, a literature search was carried out *via* online databases (e.g., PubMed, Google, and Google Scholar) reviewing the structure of POD, the reported competencies in delivering the approach, and other existing published scales relevant to Open Dialogue (See Appendix A for a categorical list of the papers and books reviewed). With the information obtained, we formed 10 potential domains on the attributes and attitudes relevant to what POD trainees should have (see Table 1). Most of these dimensions were formed under the seven principles of open dialogue created by Seikkula et al.’s (1995) team as overarching guidelines for delivering an open dialogue meeting.

**Table 1:** Ten attitude and attribute dimensions derived from the literature background and their definition.

Attitude Dimensions	
POD Principles:	Attitudes people have toward the main principles of POD, e.g., tolerating uncertainty
Peer-support role:	Acknowledging the importance of peers
POD agenda:	Agreeing that no particular objectives or plans should be made prior to meeting the client
Political and social influence:	Understanding that real-world problems, e.g., social factors may interplay with a client’s well-being.
Attribute Dimension	
A humanistic view:	Being able to talk to a client as a human with experiences rather than an ‘expert’
Trust:	Being a person that is comfortable in forming relationships and trusting others is vital.
Being present:	Not over-analyzing and offering more voice and priority toward the client.
Emotional Awareness:	Acknowledging and accepting client’s emotions is crucial.
Emotional Intelligence:	Having the ability to emphasize with client’s emotions and understand them.
Importance of Dialogue:	A mental health worker’s primary aim is to create space for dialogue.

### 2.2. Delphi round one: Interviews

As POD is a relatively new area of research, to identify the domains that are not well reported in the literature, the first round of the Delphi began with a series of semi-structured interviews.

#### 2.2.1. Participants

Eight POD practitioners that were either trainers of the POD training program or part of the Dialogue First team (Dialogue First is a non-crisis community mental health service operating in accordance with the key principles of OD within North East London NHS Foundation Trust) were invited as experts for the individual interviews (5 female) through their individual emails. The age of the interviewees ranged from 36 to 70 years (mean = 51.3 years, SD = 9.92). All the participants were from England, and their professional roles included one or more of the following: POD trainer (4), academics (3), systematic family psychotherapist (2), consultant psychologist (1), peer-support worker (2), mental health nurse (1), art therapist (1). On average, their duration of service with POD was 6.13 years (SD = 5.28).

The ethical approval of the present study is covered by the ODDESSI project, and all participants were informed that their involvement was voluntary and explicitly gave consent.

#### 2.2.2. Procedure and results

During the interview, we first provided a brief background of the current study to the practitioners. Afterward, the practitioners were asked three questions: (1) “What initially got you interested in Open Dialogue?,” (2) “Were there any changes that you experienced throughout training?,” (3) “What do you think should be considered as a measure of competence or attitude change?.” The structure of the interviews was flexible, so the nature of the follow-up questions differed between participants. In general, the interviews lasted 30 min to 1 hour. The interviews were recorded when the practitioners gave permission.

We manually transcribed and interpreted the interview content through a standardized thematic analysis by identifying and forming patterns of themes within the interview data (Braun and Clarke, 2006). Areas of the transcripts directed toward the PODACI or potential POD competencies were extracted and grouped in two separate documents. Rather than approaching the data with a pre-determined notion, we were guided by inducting reasoning and recognized common themes based on similarity, leading to the formation of 20 more domains for the PODACI (see Table 2 for the additional dimensions obtained from the interviews).

**Table 2:** Twenty attitude and attribute dimensions obtained from the interview and their definition.

Attitude Dimensions	
Trauma-informed approach:	Understanding the importance of Trauma in shaping a client’s behavior.
Family Importance:	Acknowledging the importance, a family in therapeutic context.
Losing the ‘expert role’:	Being aware of the power one has over a client, and how influential words are.
‘Nothing about them, without them’:	All discussions and plans are to be done with the client.
Personal Development:	Having a critical understanding of your own background is crucial for mental health care.
Recovery:	Understanding that recovery is a unique process that needs to acknowledge in its own way.
Client-centeredness:	All the therapy plans, and meetings should be based on the client’s input.
Tolerating uncertainty:	Experiencing silence between the mental health worker and the client has its therapeutic benefits.
Attribute Dimension	
Self-Disclosure:	Being comfortable in sharing one’s experiences to the client.
Awareness of Self-bias:	Having awareness of the prejudice and bias that one may hold.
When and What to Disclose:	Knowing when it is the right time to disclose personal information and experiences.
Active listening:	Having the ability to listen and response accordingly.
Mindfulness:	Paying attention to one’s own emotions, ideas and behaviors.
Empathy:	Acknowledging and accepting a client’s emotional status.
Accepting:	Viewing clients for who they are, and not based on their diagnosis.
Open to emotions:	Transparent with one’s own emotions and others.
Self-Compassion:	Being warm and understanding toward ourselves when we suffer, rather than ignoring our pain.
Relationship Confidence:	Feeling confident in forming new connections and bonding with new people.
Reflective of Self:	Open to feedback from both colleagues and clients.
Compassion:	Recognize the suffering of others and take action to help.

Based on the dimensions formed, we constructed items to measure each of the areas. The items would ask the trainees to rate the extent to which they agree with certain statements, from 1 (strongly disagree) to 5 (strongly agree). For example, an attribute domain like mindfulness could contain the item: ‘I pay attention to how my emotions affect my thoughts and behavior when talking with clients. How far do you agree?’. In addition, we generated reverse worded (RW) items to reduce acquiescence bias, which is the respondents’ tendency to agree with a given item regardless of its content (Zhang et al., 2016). RW items are expected to be scored lower by POD practitioners but higher with TAU professionals. For example, “I have feelings that I cannot quite identify when talking to a client” is a RW item against the attribute of mindfulness that all POD trainees should have, whereas TAU practitioners may not value as much.

In total, the first draft of the PODACI contained 30 dimensions with 167 items. The items in the draft were then assessed in the following rounds to validate their importance.

## 3. Item refinement

The second stage of the study refined the initial set of items through two rounds of questionnaires (the second the third round of Delphi) distributed to POD practitioners.

### 3.1. Delphi round two: First questionnaire

In Round Two, POD practitioners were asked to rate the importance of the 167 items generated in the first stage of the procedure, and their responses were used to refine the draft of PODACI.

#### 3.1.1. Participants

Twenty-one participants were recruited *via* an open invitation sent through the POD mailing list of the ODDESSI project. We did not set a specific selection criterion for this round as we aimed to include opinions toward the approach and training from practitioners at various stages of experience with POD. Among the participants, thirteen completed the whole round (completion rate of 65.63%), and eight dropped out in different areas of the questionnaire (data was still included). Each of the participants had either completed the POD training course prior to the study or was a trainee nearing the completion of training. Table 3 summarizes the demographic information of the participants.

**Table 3:** The demographic information of the participants in round two of Delphi, including gender, age, region of residence, professional role, and duration of service with POD.

Female gender	*n* %
	14 (66.6%)
Age	
21–30	3 (14.3%)
31–40	5 (23.8%)
41–50	6 (28.6%)
51–60	6 (28.6%)
Over 60	1 (4.76%)
Region of residence	
England	16 (76.2%)
Netherlands	3 (14.3%)
Ireland	1 (4.76%)
Wales	1 (4.76%)
Professional Role	
Academics	1 (4.76%)
Systematic family psychotherapist	1 (4.76%)
Consultant psychologists	1 (4.76%)
Peer-support worker	4 (19.1%)
Mental health nurse	3 (14.3%)
Mental health social worker	4 (19.1%)
Speech and language therapist	1 (4.76%)
Clinical psychologist	1 (4.76%)
Psychiatrists	2 (9.52%)
Doctors	1 (4.76%)
Case manager	1 (4.76%)
NHS keyworker	1 (4.76%)
Current POD trainees	3 (14.3%)
POD service time in years, mean (sd)	2.59 (1.35)

The percentage for professional roles goes above 100% because each POD practitioner has several roles.

#### 3.1.2. Procedure

The experiment was presented in a web browser using Gorilla^1^ (Anwyl-Irvine et al., 2020). After giving consent, participants were asked to provide some basic demographic information about themselves, including their gender, age, country/region of residence, professional background/role, completion of POD training, and years of services in POD. Participants were then provided with basic information about the PODACI inventory and what the items aimed to measure.

**Figure 1:**
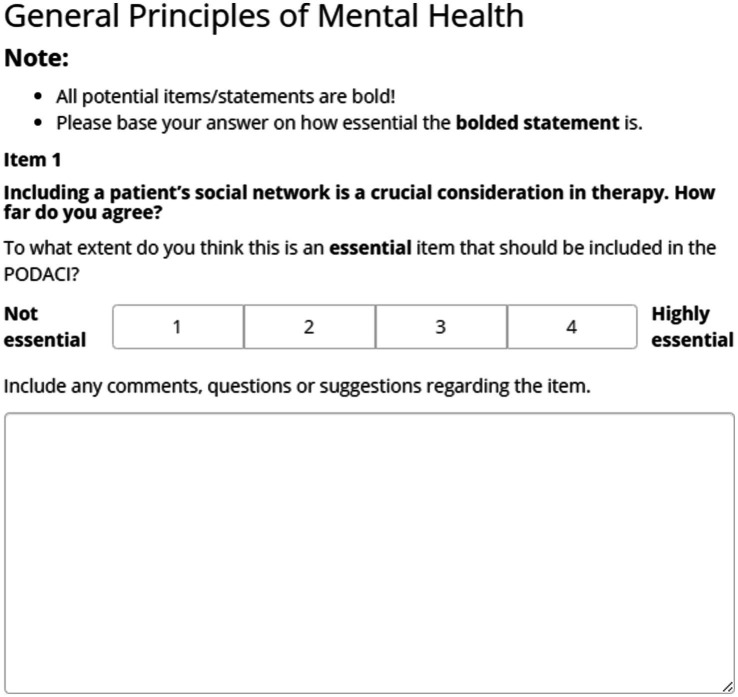
An example of the presentation of an item and the scale.

Following the instruction, the participants were presented with one item on each page and asked to rate how essential they think the item was using a 4-point Likert scale (from 1 = not essential to 4 = highly essential). Under the Likert scale, a text box was also provided for comments, questions, and suggestions. An example of how an item was presented can be found in Figure 1. At the end of the experiment, participants were given two optional open questions that asked whether the POD training changes them and what they think should be measured in PODACI.

#### 3.1.3. Analyses and results

##### 3.1.3.1. Ratings

To evaluate the consensus for each item, we calculated the median, interquartile range, and agreement level of the ratings for each item. An item was considered suitable and remained in the PODACI draft if it satisfied the following criteria: (1) the median of participants’ ratings for that item must be 3.00 or above (Mao et al., 2020), ensuring that each item is rated on a minimum of essential or higher; (2) the interquartile range must below 1.00 to indicate an agreement among the group (Raskin, 1994; Rayens and Hahn, 2000); and (3) the level of agreement must reach 85% or above. Items rated in the range of 70 to 84.9% were reconsidered in the Round 3 questionnaire, and those below a 70% rating were rejected (Mao et al., 2020; Mitchell et al., 2021). In addition, the Kendall coefficient of concordance was calculated through SPSS (IBM Corp. Released, 2019, IBM SPSS Statistics for Windows Version 26.0.) to evaluate the consensus agreement among participants (Mao et al., 2020).

Based on the criteria above, we accepted 74 items, 22 items were sent to Round Three to be reconsidered, and 71 items were deleted (see Appendix B for a detailed summary of the statistics for each item). The Kendall coefficient of concordance (W) was calculated to be 0.36 (*p* < 0.01), which indicates a fairly significant level of consensus among the participants.

##### 3.1.3.2. Open comments and questions

We manually recorded and analyzed the comments provided in the textbox for each item as well as the open question at the end of the questionnaire.

The data on the comment text box included 117 specific comments for 41 items in the attitude section and 112 comments for 71 items in the attribution section. Based on the comments, changes were made to 13 attitude items and 3 attribute items – most modifications were regarding the wording and definitions of the items as well as grammar adjustments. One item that previously fit the reconsideration criteria of ratings was also deleted due to the confusion presented in the feedback. Furthermore, while many of the reversed scored items did not meet the criteria for consensus, it can be argued that most scores given to reverse items were misinterpreted. For example, a comment for the reverse item “A professional should avoid talking about trauma unless brought up by the patient themselves” stated: “always talk about trauma if the patient has the need. Not sure if I had to score 1 or 4.” Although this participant acknowledged the importance of trauma in POD, they scored the item as 1 (non-essential) due to confusion. To compensate for this misunderstanding, seven reversed items with positive comments were reformulated as non-reverse items to be reconsidered in the next round.

Based on the responses to the open questions, we generated five more themes. Since some of the new themes overlapped with the more general topics in the pre-existing themes, we only formed two novel items for re-testing in the third round.

In total, our results suggested that 30 items needed to be reconsidered in Round Three (21 items from the ratings, 7 novel non-reverse items, and 2 items from the open questions).

### 3.2. Delphi round three: Second questionnaire

The third round consisted of a new questionnaire that measured the relevance of 30 items that were deemed necessary to be re-tested.

#### 3.2.1. Participants

We contacted the 13 participants who had completed the second round for further re-testing and 10 participants responded (8 female). The age of the participants ranged from 29 to 60 years (mean = 45.78 years, SD = 12.22). All the participants are from England, and their professional roles included peer-support worker (3), mental health nurse (4), social worker (1), clinical psychologist (1), and speech and language therapist (1). Three of the participants were current POD trainees, and on average, their duration of service with POD was 1.44 years (SD = 1.22).

#### 3.2.2. Procedure

The procedure followed that of round two (first questionnaire). The practitioners rated how essential the items were based on the 4-point Likert scale. Uniquely to round three, each item was presented with a group aggregated rating based on the previous round to promote more consideration in the individual’s answers.

#### 3.2.3. Analyses and results

Using the same consensus criteria as round two, the reconsidered items were either accepted (85% agreement level or above) or deleted (anything below 85% agreement level), as there were no more rounds for re-assessment. The Kendall coefficient of concordance was also calculated.

Overall, the Kendal coefficient of concordance (W) for this round was 0.28 (*p* < 0.01), indicating a fairly significant level of group consensus. Three items were deleted because they did not satisfy the consensus criteria and 27 items were accepted and added to the PODACI draft (see Appendix C for a detailed summary of the statistics for each item).

In total, the second draft of the PODACI now contained 102 items (75 items from round two and 27 items from round three).

## 4. Inventory finalization

### 4.1. Delphi round four: Group interview

The fourth and final round of our Delphi procedure aimed to finalize the structure and content of the PODACI through a focus group interview with POD trainers as they have extensive experiences with the training program’s goals, procedure, and its effect on trainees (some participated in the previous rounds).

#### 4.1.1. Participants

Four POD trainers were invited to the group interview (3 participated in the first round, 2 female). The age range of the trainees was from 49 to 70 years (mean = 58.5 years, *SD* = 8.66). Three of the trainers resided in England and one was from Norway. Their professional role included one or more of the following; POD trainers (4), academics (1), systematic family psychotherapist (2), and consultant psychologists (1). The trainers’ average duration of POD service was 13.5 years (SD = 5.26).

#### 4.1.2. Procedure and results

Before the interview, the panelists had to complete a questionnaire based on the second draft of the PODACI. The questionnaire asked the panelists to rate how essential each item was on a four-point Likert scale similar to round two and three. There were no open-based questions or group aggregated ratings. Items that received a median rating of 3.5 and a level of agreement above 85% were automatically kept in PODACI. Any items whose ratings did not reach a median of 3.50 and a level of agreement between 85 and 100% were discussed in the group interview for further clarification. Items with a median rating below 2.00 were removed. A higher selection criterion (i.e., median rating above 3.50) was necessary to identify any minor discrepancies within items.

Based on the ratings in the questionnaire, a word document was made of items that required clarification and sent to the participants. During the focus group interview, we read out the items of interest, and the group covered any emerging differences of opinions. Once a verbal agreement was evident on a particular item, the feedback was applied to the PODACI, forming the final draft of the inventory.

Findings from the fourth round of questionnaires identified 49 items that met the consensus criteria, six items that were removed due to a low score, and 47 items considered for further discussion in the group interview (see Appendix D for detailed statistics for each item). The Kendal coefficient of concordance (W) for the questionnaire was 0.54 (*p* < 0.01), indicating a strong agreement. Based on the group meeting on the 47 reconsidered items, 19 items were changed, 20 items were deleted, and eight items remained. In total, the final version of the PODACI contained 76 items summarized in Table 4.

**Table 4:** The final version of PODACI.

Peer-supported open dialogue attitude and competence inventory (PODACI)
Attitude dimensions
General principles of mental health care
1. Clients should always be allowed to invite their social network to their meetings. How far do you agree?
2. Having the same team offer continuous care to a client over months and potentially years is more effective than care that is delivered consecutively by multiple specialized teams. How far do you agree?
3. The client should generally be allowed to decide the timing of the next meeting. How far do you agree?
4. Most of what is considered symptoms of mental illness, is actually meaningful behavior. How far do you agree?
5. The primary goal of mental health care should be to increase the agency of the client. How far do you agree?
6. The help offered should be dictated by the needs of the client. How far do you agree?
7. Being open about your feelings and experiences is a necessary skill in mental health treatment. How far do you agree?
8. Mental health care should place emphasis on the client’s words and emotions present in the meeting, rather than the diagnosis, when considering treatment and medication. How far do you agree?
Trauma
9. Clients should be supported to talk about the possible role of trauma, abuse, and neglect in the development of their mental health issues. How far do you agree?
10. What has happened to a client shapes their mental health wellbeing in later life. How far do you agree?
11. The way most mental health services are currently delivered can easily be re-traumatizing for clients. How far do you agree?
12. Most of what is diagnosed as mental illness is the result of trauma. How far do you agree?
Recovery
13. For some forms of mental illness, recovery is not possible. How far do you agree? (REVERSE)
14. Experiencing setbacks is a normal part of a client’s recovery. How far do you agree?
15. Clients have different ways in how they recover from mental illnesses. How far do you agree?
16. All people with serious mental illnesses can strive for recovery. How far do you agree?
17. Clients are ‘experts by experience’ who play the most important role in their own recovery. How far do you agree?
Client-centeredness
18. One of the practitioner’s main function is to try to convey to the client that they are listening and are accepting of the client’s feelings and attitudes. How far do you agree?
19. A specific and thorough diagnosis is essential for effective outcomes in mental health care. How far do you agree? (REVERSE)
20. When in a meeting with a client, what is important is your ability to ‘be with them’ rather than ‘doing something to them’. How far do you agree?
Tolerating silence and uncertainty
21. Tolerating silence or uncertainty in a client meeting can lead to beneficial outcomes. How far do you agree?
22. If a client wishes to spend time in silence, they should be allowed. How far do you agree?
23. Tolerating silence between you and the client has therapeutic benefits. How far do you agree?
Having no agenda
24. Having no fixed objectives when meeting clients, allows more free exchange with the client and creates more meaningful experiences. How far do you agree?
25. Rather than focusing on the client’s problems, practitioners should listen for meaningful expressions and strive to help the client make sense of what they are feeling. How far do you agree?
Peer support worker
26. Peer support should be offered as part of all mental health care services. How far do you agree?
27. In mental health teams, peers (persons with lived experience) are of equal status and value of opinion. How far do you agree?
28. Peers (persons with lived experience) should be involved at every level of service delivery. How far do you agree?
29. Peers (persons with lived experience) provide a different experiential level of understanding of a client’s distress, that is important to include in mental health care. How far do you agree?
Having no ‘expert’ role
30. The primary role of a practitioner is to create a safe space where the client and their network feel free to speak. How far do you agree?
31. Practitioners are there to support the mutual learning between themselves and the client, both sides learn from each other. How far do you agree?
32. Saying less as a practitioner rather than more is an effective way of treatment care. How far do you agree?
Family importance
33. Including and supporting a client’s social network as soon as possible, is an important part of mental health care. How far do you agree?
‘Nothing about them, without them’
34. Practitioners should never talk about a client without the client being present. How far do you agree?
35. All issues and solutions should be openly discussed with the client for effective therapeutic treatment. How far do you agree?
36. Practitioners should not decide on any plans before meeting the client. How far do you agree?
Personal development
37. It would benefit me to understand my own life history in order to be of help to others. How far do you agree?
38. My personal values and attitudes have a major impact on how I communicate with my clients. How far do you agree?
Political and social influence
39. It is important to consider the political and social factors that may negatively impact a client. How far do you agree?
Attribute dimensions
Mindfulness
40. I pay attention to how my emotions affect my thoughts and behavior when talking with clients. How far do you agree?
41. When I have distressing thoughts or images during my meeting with a client, I make an effort to “step back” and be aware of the thoughts or images without getting taken over by them. How far do you agree?
42. Having a daily mindfulness practice can be an important part of my work. How far do you agree?
43. I endeavor to always be aware of the feelings that I experience when talking with the client. How far do you agree?
Self-compassion
44. Self-care is an important part of my professional work. How far do you agree?
45. When I feel down in some way, I try to remind myself these feelings are shared by most people in the service, and this may be a way that I can establish a connection with my clients. How far do you agree?
46. I feel comfortable expressing my sadness and worries in front of colleagues and clients. How far do you agree?
Emotional awareness
47. Responding to the client emotionally is often the most important work done in meetings. How far do you agree?
48. I give less primacy to the ideas of looking for a diagnosis or a solution, and instead, focus on the client and what is happening in their lives. How far do you agree?
Awareness of self-Bias
49. I can recognize my own biases that could negatively impact a client. How far do you agree?
50. Self-work is an important part of my development. How far do you agree?
51. Learning to know myself better is an important goal for my professional development. How far do you agree?
Self-disclosure
52. I feel confident in opening up and sharing my life experiences with clients and colleagues. How far do you agree?
53. I am able to discuss sensitive things about myself with the client if it is suitable and safe for both sides. How far do you agree?
Knowing when and what to self-disclose
54. I can disclose my own personal experiences to the client when I feel it would be beneficial for the client. How far do you agree?
55. It is sometimes better to stay quiet than to talk. How far do you agree?
Compassion
56. When a client is upset, I try to stay open to their feelings rather than avoid them. How far do you agree?
A humanistic approach
57. People often need a fellow human being to relate and talk to. How far do you agree?
58. I am able to care deeply about every client I work with. How far do you agree?
59. Just being a fellow human being is sometimes the most important thing a practitioner can offer a person in crisis. How far do you agree?
60. A practitioner is a human first, and then they are a human with some expertise. How far do you agree?
61. Being authentic and honest is an important skill that I try to practice on a daily basis. How far do you agree?
Giving away power
62. I am able to listen to my client, without stepping in and ‘wanting to fix the problem’. How far do you agree?
63. I feel confident in letting the client lead the conversations and meetings. How far do you agree?
64. I am able to filter out ideas of diagnosis, solutions and stay attentive to the client. How far do you agree?
65. It is important that I understand how my position of power and privilege influences my relationships with clients. How far do you agree?
Accepting
66. I view clients for who they are and not based on their diagnosis. How far do you agree?
67. I take time to understand the client and their experiences. How far do you agree?
68. I am good at understanding an individual’s perspectives. How far do you agree?
Reflective of one-self
69. When I make mistakes in a meeting, I apologize to the client. How far do you agree?
70. There are always areas I can work to improve. How far do you agree?
71. I am open to feedback from my colleagues and clients. How far do you agree?
Tolerating uncertainty and silence
72. Tolerating silence between myself and the client is stressful (REVERSE). How far do you agree?
73. I can keep an open mind and allow space and time for a client to reflect. How far do you agree?
Relationships
74. I give a lot of attention to the family that surrounds my client and their relationship. How far do you agree?
Meeting priorities with clients
75. One of my primary goals is to facilitate an emotional exchange between the client and their network. How far do you agree?
Self-reflection
76. I am willing to watch myself back on video and reflect on areas that I may need to work on. How far do you agree?

When in use, trainees would be asked to give a rating based on a five-point Likert scale from 1 (Strongly disagree) to 5 (Strongly agree).

## 5. Discussion

The present study aimed to create an inventory that can assess how prepared POD trainees are and the efficacy of the training course. With a four-round modified Delphi procedure, the current study generated the PODACI with 76 items.

All the items included in PODACI had a good consensus with a median range score of 3.00 or above in round two and round three and 3.50 or above in round four, an interquartile range from 0.00 to 1.00, and an agreement level over 85%. The Kendall coefficient of concordance (W) used to assess the agreement among the participants (Gearhart et al., 2013) was 0.36 in round two, 0.28 in round three, and 0.54 in round four, which showed fair to good level of agreement among participants (Gearhart et al., 2013; Mao et al., 2020).

The items in the PODACI have been specifically tailored to what POD trainees should present at the end of training. Specifically, the PODACI covered the 12 key elements of fidelity to dialogic practice (Olson et al., 2014), a wide range of attitudes toward the general principles of POD (Seikkula et al., 1995), as well as factors like peer-support and mindfulness that were reported to be essential in practice (Hopfenbeck, 2015; Razzaque and Stockmann, 2016; Jackson and Thorley, 2021). This highly enriched instrument opens up for a wide range of possibilities for POD research and application within and outside of the United Kingdom, as discussed below.

First, the PODACI provided a way to measure attitudinal and competency factors related to the treatment integrity of POD. With the instrument, OD researchers and trainers can examine the developmental changes within individual trainees, ensuring they have developed the necessary competence and attitudes to deliver POD as intended. In addition, PODACI offers the potential for large-scale studies with quantitative data that may be more reliable and comparable with pre-existing findings around the world. Practically, the quantitative research made available by the PODACI may generate straightforward evidence on the benefits of the POD training that can be presented to the service development managers and policymakers. Combining PODACI with previous qualitative studies could offer a more comprehensive view of POD training to the NHS, promoting the implementation of POD in the system.

Second, the PODACI could facilitate the development of training courses provided to future POD practitioners. At the moment, there were no available tools or procedures to systematically examine the efficacy of the training, so whether the courses were enough for trainees to practice POD appropriately was unclear. Examining POD trainees’ responses to PODACI before and after training could (1) show which OD dimensions the training helped to improve the most or the least on a group level, and (2) inform the trainers what may be harder or easier to apprehend for each individual trainee. This information provided by PODACI could be used to advance the courses in general and to modify the training base on individual needs, which could lead to better trained practitioners.

Last but not the least, PODACI could be generalized to areas outside of POD as it covers a wide range of values and attributes necessary for general mental health practice. Within the United Kingdom, the NHS has presented a long-term forward plan (Alderwick and Dixon, 2019) that aims to review and advance the competencies of all mental health treatments available. PODACI could be used as a format for therapy approaches that shares some of the same essential qualities as POD to develop inventories in treatment integrity, such as systematic family therapy. In this way, practitioners could also compare various training schemes in family therapy, identifying the benefits and disadvantages of each and improve them accordingly.

While we need to acknowledge that the current study has a relatively small sample size, all of our participants are highly knowledgeable in POD and POD training. In a Delphi study, one of the most important considerations in sample collection is the selection of participants who are knowledgeable in the field of the study (Grisham, 2009). Skulmoski et al. (2007) validated the stability of response characteristics in a small panel and argued that a limited number of experts with similar training and knowledge would still yield reliable results, which is the case of the current study. Furthermore, more than half of the practitioners spent over 2 hours completing the two rounds of questionnaires (estimated time of completion is around 30–40 min), and the majority of interviews lasted as long as an hour, indicating that the panelists in this study were motivated in giving their best effort to help the development of the PODACI. Such enthusiasm could reinforce the content validity of the PODACI (Goodman, 1987).

## 6. Conclusion

In conclusion, the current paper established an inventory that investigates the changes in POD trainees’ attitudes and measures the general effectiveness of the current training course. The inventory consists of 27 domains and 76 items. A panel of POD practitioners and trainers reached a consensus on all the items that were included in this scale, while items with a low consensus throughout the Delphi rounds were removed. This study is a first step to fully develop and validate the PODACI. The next stage for the PODACI would be to test the inventory further on POD trainees, validating the instrument for its psychometric quality, and examining the reliability and validity of the items included. Further research also needs to assess the relationship between the attitudes and attribute items with OD principles and treatment outcomes, helping understand how certain types of items are related to a successful OD delivery. Additionally, pilot studies done on TAU and POD practitioners are required to see if POD practitioners score differently than TAU professionals (with POD practitioners expected to score higher) and if the inventory functions as intended. Once verified, researchers, POD trainers, and policymakers will have a working inventory to use.

## Data availability statement

The raw data supporting the conclusions of this article will be made available by the authors, without undue reservation.

## Ethics statement

Ethical review and approval was not required for the study on human participants in accordance with the local legislation and institutional requirements. Written informed consent for participation was not required for this study in accordance with the national legislation and the institutional requirements.

## Author contributions

VF responsible for the conceptualization and design of the manuscript, collected the data, performed the data analyses, and drafted the manuscript. JS drafted the initial version of the manuscript, reviewed, and revised the work. MH responsible for the conceptualization and design of the manuscript, reviewed, and commented the drafts. All authors contributed to the article and approved the submitted version.

## Conflict of interest

The authors declare that the research was conducted in the absence of any commercial or financial relationships that could be construed as a potential conflict of interest.

## Publisher’s note

All claims expressed in this article are solely those of the authors and do not necessarily represent those of their affiliated organizations, or those of the publisher, the editors and the reviewers. Any product that may be evaluated in this article, or claim that may be made by its manufacturer, is not guaranteed or endorsed by the publisher.

## Footnotes

^1^ www.gorilla.sc
