# Supplementary material for: Development of the peer-supported open dialogue attitude and competence inventory for practitioners: A Delphi study
Source: Front Psychol. 2023 Mar 30;14:1059103. doi: 10.3389/fpsyg.2023.1059103 (PMC10097924; doi:10.3389/fpsyg.2023.1059103)
Supplement: Supplementary file 1 [file Table_1.DOCX]

Supplementary Material

Appendix A. Literature Review of papers and books included

OD Training:

Razzaque, R. (2019). Dialogical Psychiatry: A Handbook For The Teaching And Practice Of Open Dialogue (1st ed.)

OD & POD:

Bergström, T., Seikkula, J., Alakare, B., Mäki, P., Köngäs-Saviaro, P., Taskila, J. J., ... & Aaltonen, J. (2018). The family-oriented open dialogue approach in the treatment of first-episode psychosis: Nineteen–year outcomes. *Psychiatry research*, *270*, 168-175.

Freeman, A. M., Tribe, R. H., Stott, J. C., & Pilling, S. (2019). Open dialogue: a review of the evidence. *Psychiatric Services*, *70*(1), 46-59.

Gordon, C., Gidugu, V., Rogers, E. S., DeRonck, J., & Ziedonis, D. (2016). Adapting open dialogue for early-onset psychosis into the US health care environment: A feasibility study. *Psychiatric Services*, *67*(11), 1166-1168.

Granö, N., Kallionpää, S., Karjalainen, M., Salmijärvi, L., Roine, M., & Taylor, P. (2016). Declines in suicidal ideation in adolescents being treated in early intervention service. *Psychosis*, *8*(2), 176-179.

Hopfenbeck, M. (2015). Peer-supported open dialogue. *Context*, *138*, 29-31.

Olson, M., Seikkula, J., & Ziedonis, D. (2014). The key elements of dialogic practice in open dialogue: Fidelity criteria. *Worcester: University of Massachusetts Medical School*.

Ong, B., Barbara‐May, R., Brown, J. M., Dawson, L., Gray, C., McCloughen, A., ... & Buus, N. (2019). Open dialogue: Frequently asked questions. *Australian and New Zealand Journal of Family Therapy*, *40*(4), 416-428.

Pavlovic, R. Y., Pavlovic, A., & Donaldson, S. (2016). Open Dialogue for psychosis or severe mental illness. *The Cochrane Database of Systematic Reviews*, *2016*(10).

Razzaque, R. (2015). Mindfulness and open dialogue: A common foundation and a common practice. *Context*, *137*, 45.

Razzaque, R., & Stockmann, T. (2016). An introduction to peer-supported open dialogue in mental healthcare. *BJPsych Advances*, *22*(5), 348-356.

Seikkula, J., Aaltonen, J., Alakare, B., Haarakangas, K., Keränen, J., & Lehtinen, K. (2006). Five-year experience of first-episode nonaffective psychosis in open-dialogue approach: Treatment principles, follow-up outcomes, and two case studies. *Psychotherapy research*, *16*(02), 214-228.

Seikkula, J., Alakare, B., & Aaltonen, J. (2011). The comprehensive open-dialogue approach in Western Lapland: II. Long-term stability of acute psychosis outcomes in advanced community care. *Psychosis*, *3*(3), 192-204.

Seikkula, J., Alakare, B., Aaltonen, J., Holma, J., Rasinkangas, A., & Lehtinen, V. (2003). Open dialogue approach: Treatment principles and preliminary results of a two-year follow-up on first episode schizophrenia. *Ethical human sciences and services*, *5*(3), 163-182.

Relevant Scales:

Austin, J. L. (2020). *Development and Validation of the Counselors' Emotional Awareness Scale (C-EAS)* (Doctoral dissertation, The University of North Carolina at Greensboro).

Baer, R. A., Smith, G. T., Lykins, E., Button, D., Krietemeyer, J., Sauer, S., ... & Williams, J. M. G. (2008). Construct validity of the five facet mindfulness questionnaire in meditating and nonmeditating samples. *Assessment*, *15*(3), 329-342.

Borkin, J. R., Steffen, J. J., Ensfield, L. B., Krzton, K., Wishnick, H., Wilder, K., & Yangarber, N. (2000). Recovery attitudes questionnaire: Development and evaluation. *Psychiatric Rehabilitation Journal*, *24*(2), 95.

Breunlin, D. C., Schwartz, R. C., Krause, M. S., & Selby, L. M. (1983). Evaluating family therapy training: The development of an instrument. *Journal of Marital and Family Therapy*, *9*(1), 37-47.

Bybee, B., & Frost, J. (2017). Active Listening Attitude Scale (ALAS) (Mishima, Kubota, & Nagata, 2000). *The Sourcebook of Listening Research: Methodology and Measures*, 167-173.

Frazier, M. L., Johnson, P. D., & Fainshmidt, S. (2013). Development and validation of a propensity to trust scale. *Journal of Trust Research*, *3*(2), 76-97.

Gu, J., Baer, R., Cavanagh, K., Kuyken, W., & Strauss, C. (2020). Development and psychometric properties of the Sussex-Oxford compassion scales (SOCS). *Assessment*, *27*(1), 3-20.

Hojat, M., Mangione, S., Nasca, T. J., Cohen, M. J., Gonnella, J. S., Erdmann, J. B., ... & Magee, M. (2001). The Jefferson Scale of Physician Empathy: development and preliminary psychometric data. *Educational and psychological measurement*, *61*(2), 349-365.

Jacobson, C., Hill, R. M., Pettit, J. W., & Miranda, R. (2015). The Measure of Verbally Expressed Emotion: Development and factor structure of a scale designed to assess comfort expressing feelings to others. *Journal of Psychopathology and Behavioral Assessment*, *37*(2), 358-369.

Tapia, M. (2001). Measuring emotional intelligence. *Psychological Reports*, *88*(2), 353-364.

Mindfulness:

Amanda Shallcross, N. D., Lu, N. Y., & Hays, R. D. Evaluation of the Psychometric Properties of the Five Facet of Mindfulness Questionnaire.

Baer, R. A., Smith, G. T., Hopkins, J., Krietemeyer, J., & Toney, L. (2006). Using self-report assessment methods to explore facets of mindfulness. *Assessment*, *13*(1), 27-45.

Beach, M. C., Roter, D., Korthuis, P. T., Epstein, R. M., Sharp, V., Ratanawongsa, N., ... & Saha, S. (2013). A multicenter study of physician mindfulness and health care quality. *The Annals of Family Medicine*, *11*(5), 421-428.

Bohlmeijer, E., Ten Klooster, P. M., Fledderus, M., Veehof, M., & Baer, R. (2011). Psychometric properties of the five facet mindfulness questionnaire in depressed adults and development of a short form. *Assessment*, *18*(3), 308-320.

Davis, D. M., & Hayes, J. A. (2011). What are the benefits of mindfulness? A practice review of psychotherapy-related research. *Psychotherapy*, *48*(2), 198.

Iani, L., Lauriola, M., & Cafaro, V. (2020). The assessment of mindfulness skills: the “what” and the “how”. *Journal of Mental Health*, *29*(2), 144-151.

Lilja, J. L., Falkenström, F., Zelleroth, C., Jacobson, E., Risberg, S., Nissling, L., & Weineland, S. (2020). Psychometric properties and validation of the Swedish Five Facet Mindfulness Questionnaire in a clinical and non‐clinical sample among meditators and non‐meditators. *Scandinavian Journal of Psychology*, *61*(3), 369-379.

Lilja, J. L., Frodi-Lundgren, A., Hanse, J. J., Josefsson, T., Lundh, L. G., Sköld, C., ... & Broberg, A. G. (2011). Five facets mindfulness questionnaire—reliability and factor structure: a Swedish version. *Cognitive behaviour therapy*, *40*(4), 291-303.

Pratscher, S. D., Wood, P. K., King, L. A., & Bettencourt, B. (2019). Interpersonal mindfulness: Scale development and initial construct validation. *Mindfulness*, *10*(6), 1044-1061.

Razzaque, R. (2015). Mindfulness and open dialogue: A common foundation and a common practice. *Context*, *137*, 45.

Empathy:

Alcorta-Garza, A., San-Martín, M., Delgado-Bolton, R., Soler-González, J., Roig, H., & Vivanco, L. (2016). Cross-validation of the Spanish HP-version of the jefferson scale of empathy confirmed with some cross-cultural differences. Frontiers in Psychology, 7, 1002.

Hojat, M. (2007). Empathy in patient care: Antecedents, development, measurement, and outcomes.

Hojat, M. (2016). The Jefferson Scale of Empathy. In Empathy in Health Professions Education and Patient Care (pp. 83-128). Springer, Cham.

Hojat, M., Mangione, S., Nasca, T. J., Cohen, M. J., Gonnella, J. S., Erdmann, J. B., ... & Magee, M. (2001). The Jefferson Scale of Physician Empathy: development and preliminary psychometric data. Educational and psychological measurement, 61(2), 349-365.

Montanari, P., Petrucci, C., Russo, S., Murray, I., Dimonte, V., & Lancia, L. (2015). Psychometric properties of the J efferson S cale of E mpathy‐H ealth P rofessional S tudent's version: An I talian validation study with nursing students. Nursing & health sciences, 17(4), 483-491.

Ward, J., Schaal, M., Sullivan, J., Bowen, M. E., Erdmann, J. B., & Hojat, M. (2009). Reliability and validity of the Jefferson Scale of Empathy in undergraduate nursing students. Journal of Nursing Measurement, 17(1), 73.

Compassion:

Burnell, L., & Agan, D. L. (2013). Compassionate care: Can it be defined and measured? The development of the Compassionate Care Assessment Tool. *International Journal of Caring Sciences*, *6*(2), 180-187.

Gu, J., Baer, R., Cavanagh, K., Kuyken, W., & Strauss, C. (2020). Development and psychometric properties of the Sussex-Oxford compassion scales (SOCS). *Assessment*, *27*(1), 3-20.

Strauss, C., Taylor, B. L., Gu, J., Kuyken, W., Baer, R., Jones, F., & Cavanagh, K. (2016). What is compassion and how can we measure it? A review of definitions and measures. *Clinical psychology review*, *47*, 15-27.

Self-Compassion:

Neff, K. D. (2003). The development and validation of a scale to measure self-compassion. *Self and identity*, *2*(3), 223-250.

Neff, K. D. (2016). The self-compassion scale is a valid and theoretically coherent measure of self-compassion. *Mindfulness*, *7*(1), 264-274.

Raes, F., Pommier, E., Neff, K. D., & Van Gucht, D. (2011). Construction and factorial validation of a short form of the self‐compassion scale. *Clinical psychology & psychotherapy*, *18*(3), 250-255.

Transparency:

Jacobson, C., Hill, R. M., Pettit, J. W., & Miranda, R. (2015). The Measure of Verbally Expressed Emotion: Development and factor structure of a scale designed to assess comfort expressing feelings to others. *Journal of Psychopathology and Behavioral Assessment*, *37*(2), 358-369.

Self-Awareness:

Rasheed, S. P., Sundus, A., Younas, A., Fakhar, J., & Inayat, S. (2021). Development and testing of a measure of self-awareness among nurses. *Western journal of nursing research*, *43*(1), 36-44.

Emotional Intelligence:

Tapia, M. (2001). Measuring emotional intelligence. *Psychological Reports*, *88*(2), 353-364.

Active Listening:

Busiol, D. (2018). The Development of a Listening Scale. *Research on Social Work Practice*, *28*(8), 973-980.

Bybee, B., & Frost, J. (2017). Active Listening Attitude Scale (ALAS) (Mishima, Kubota, & Nagata, 2000). *The Sourcebook of Listening Research: Methodology and Measures*, 167-173.

Mishima, N., Kubota, S., & Nagata, S. (2000). The development of a questionnaire to assess the attitude of active listening. *Journal of Occupational Health*, *42*(3), 111-118.

Emotional Awareness:

Austin, J. L. (2020). *Development and Validation of the Counselors' Emotional Awareness Scale (C-EAS)* (Doctoral dissertation, The University of North Carolina at Greensboro).

Recovery Attitude:

Bedregal, L. E., O'Connell, M., & Davidson, L. (2006). The Recovery Knowledge Inventory: assessment of mental health staff knowledge and attitudes about recovery. *Psychiatric rehabilitation journal*, *30*(2), 96.

Borkin, J. R., Steffen, J. J., Ensfield, L. B., Krzton, K., Wishnick, H., Wilder, K., & Yangarber, N. (2000). Recovery attitudes questionnaire: Development and evaluation. *Psychiatric Rehabilitation Journal*, *24*(2), 95.

Gudjonsson, G. H., Webster, G., & Green, T. (2010). The recovery approach to care in psychiatric services: staff attitudes before and after training. *The Psychiatrist*, *34*(8), 326-329.

Salgado, J. D., Deane, F. P., Crowe, T. P., & Oades, L. G. (2010). Hope and improvements in mental health service providers' recovery attitudes following training. *Journal of Mental Health*, *19*(3), 243-248.

Appendix B. Item Statistics for the second-round questionnaire

| Note: * = not meet the criteria of consensus | | | | |
| --- | --- | --- | --- | --- |
| **Domains** | **Attitude Items** | **Median** | **Inter-quartile Range** | **Level of Agreement** |
| **General Principles of Mental Health Care** | 1. Including a patient’s social network is a crucial consideration in therapy. | 4.00 | 0 | 1.00 |
|  | 1. Providing a rapid response within 24 hours at the point of referral is an effective principle to adhere to in mental health care. | 3.00 | 1.00 | 0.90 |
|  | 1. Having the same team members responsible for the care of a patient is more effective to their treatment. | 4.00 | 1.00 | 1.00 |
|  | 1. Mental health treatment should be flexible, allowing patients to decide the number of days and week that are necessary to meet up. | 4.00 | 1.00 | 0.95 |
|  | 1. If a person is psychotic it is often best to administer medication and wait until the person is stable prior to starting therapy (REVERSE). | 1.00* | 2* | 0.33* |
|  | 1. If a person is hearing voices, it is important to help the person understand that the voices are not real (REVERSE). | 1.00* | 1.00 | 0.14* |
|  | 1. Risk assessment should be the primary issue when working with suicidal or aggressive patients (REVERSE). | 2.00* | 1.00 | 0.43* |
|  | 1. Most of what is considered symptoms of mental illness, is actually meaningful behaviour. | 4.00 | 1.00 | 0.95 |
|  | 1. It is important to understand that patients in crisis can often be manipulative, attention-seeking or destructive (REVERSE). | 1.00* | 1.00 | 0.10* |
|  | 1. The primary goal of therapy should be to increase the agency of the patient. | 3.00 | 1.00 | 0.86 |
|  | 1. The initial goal of therapy should be to reduce the patients’ symptoms (REVERSE). | 2.00* | 1.00 | 0.33* |
|  | 1. What you offer and how you help all depends on the needs of the patient. | 4.00 | 1.00 | 0.90 |
|  | 1. Being open about your feelings and experiences is a necessary skill in therapy | 4.00 | 1.00 | 1.00 |
| **Trauma** | 1. Trauma is something that needs to be explored in all patient meetings | 4.00 | 1.00 | 1.00 |
|  | 1. The experiences a patient has shapes a lot of their later life. | 4.00 | 0 | 0.90 |
|  | 1. Most of what is diagnosed as mental illness is the result of trauma | 3.00 | 1.00 | 0.81 |
|  | 1. The way mental health services are currently delivered can often be re-traumatizing for patients. | 4.00 | 1.00 | 0.95 |
|  | 1. A professional should avoid talking about trauma unless brought up by the patient themselves (REVERSE). | 2.00* | 2* | 0.24* |
|  | 1. Mentioning trauma may make the situation worse and hinder recovery (REVERSE) | 1.00* | 1.00 | 0.24* |
| **Recovery** | 1. Recovering from a mental illness is possible no matter what the situation is. | 4.00 | 1.00 | 0.90 |
|  | 1. Patients in recovery sometimes have setbacks | 4.00 | 0 | 0.86 |
|  | 1. Patients have different ways in how they recover from mental illnesses. | 4.00 | 1.00 | 0.95 |
|  | 1. All people with serious mental illnesses can strive for recovery | 4.00 | 1.00 | 0.90 |
|  | 1. Patients are ‘experts by experience’ who play a role in their own recovery | 4.00 | 0 | 1.00 |
|  | 1. Recovery is only for individuals, not for families or teams (REVERSE) | 1.00* | 1.00 | 0.24* |
|  | 1. Patients rarely have anything useful to say about their treatment (REVERSE) | 1.00* | 1.00 | 0.24* |
| **Client-Centered-ness** | 1. A thorough diagnosis is unnecessary for effective counselling | 3.00 | 1.00 | 0.76 |
|  | 1. The counsellor should always be the one to determine when the next meeting with the patient is done (REVERSE) | 1.00* | 2* | 0.29* |
|  | 1. One of the professional’s main function is to try to convey to the patient that they are listening and area accepting of the person's feeling and attitudes. | 4.00 | 1.00 | 0.90 |
|  | 1. If a patient wants to discontinue the meetings, they are allowed to do so. | 2.00* | 2* | 0.90 |
|  | 1. The professional should be objective and impersonal in their relationship with the patients (REVERSE) | 2.00* | 2* | 0.38* |
|  | 1. It is unnecessary for the counsellor to obtain a clear picture of the nature and origins of the patient’s problem before they can help them. | 2.00* | 2* | 0.43* |
| **Tolerating silence and uncertainty** | 1. Tolerating silence or uncertainty in a patient meeting can lead to beneficial outcomes. | 4.00 | 0 | 1.00 |
|  | 1. If a patient wishes to spend time in silence, they should be allowed. | 4.00 | 0 | 0.90 |
|  | 1. A therapist should avoid silence with the therapist as that only makes things awkward (REVERSE) | 1.00* | 1.00 | 0.24* |
|  | 1. Professionals should always have a plan when meeting a patient and be aware of the situation (REVERSE) | 2.00* | 2* | 0.33* |
| **Having no agenda** | 1. Having ‘no fixed objectives’ in therapy, allows more free exchange with the patient and creates more meaningful experiences. | 4.00 | 0 | 1.00 |
|  | 1. Rather than focusing on the problem, mental health worker should listen out for meaningful comments and strive to make sense of what the patient feels – ‘normalizing discourse’. | 4.00 | 0 | 1.00 |
| **Peer support worker** | 1. Peer support is an important facilitator of individual mental health recovery. | 4.00 | 1.00 | 0.95 |
|  | 1. Peers (persons with lived experience) should be equal members of mental health teams | 4.00 | 1.00 | 1.00 |
|  | 1. Peers (persons with lived experience) often understand the patient’s emotional distress better than the other professionals | 3.00 | 1.00 | 0.76 |
|  | 1. Peers (persons with lived experience) should be involved at every level of service delivery | 4.00 | 1.00 | 1.00 |
|  | 1. A mental health worker is not there to dominate a patient with their ideas but is simply there to create a safe space where the client can talk. | 4.00 | 0 | 1.00 |
|  | 1. Mental health workers are there to support mutual learning between them and the patient, both sides can learn from each other. | 4.00 | 0 | 0.95 |
|  | 1. Emphasis should be placed on the mental health workers and their opinions (REVERSE) | 1.00* | 2* | 0.29* |
|  | 1. Saying less as a therapist rather than more is an effective way of treatment care. | 3.00 | 1.00 | 0.90 |
| **Family importance** | 1. Understanding a patient’s connections in a family is an important step in therapy. | 4.00 | 1.00 | 0.90 |
|  | 1. Including one’s family in a therapy meeting will cause more problems than benefits (REVERSE) | 1.00* | 2* | 0.29* |
| **‘Nothing about them, without them’** | 1. Professionals should never talk about a patient without them being present. | 3.00 | 1.00 | 0.86 |
|  | 1. All issues and solutions should be openly discussion with the patient for effective therapeutic treatment. | 4.00 | 1.00 | 1.00 |
|  | 1. It is often necessary to discuss a patient case with colleagues prior to the therapy, in order to understand what is best for the patient (REVERSE) | 2.00* | 2* | 0.29* |
| **Personal development** | 1. My personal values and attitudes have a major impact on how I do my work | 4.00 | 1.00 | 0.81 |
|  | 1. I need to understand my own life history in order be of help to others | 4.00 | 1.00 | 1.00 |
|  | 1. Having a critical understanding my own cultural background helps me provide competent care for persons with cultural backgrounds different from my own | 3.00 | 1.00 | 0.90 |
|  | 1. Self-development is something that is not necessary when providing mental health care for others (REVERSE) | 1.00* | 1.00 | 0.19* |
|  | 1. It is important to keep my personal life separate from my professional work (REVERSE) | 2.00* | 1.00 | 0.24* |
| **Political and social influence** | 1. It is important to consider the political and social factors that may negatively impact the patient. | 4.00 | 1.00 | 0.95 |
|  | 1. Professionals should avoid discussing social and political issues in meetings (REVERSE). How far do you agree? | 1.00* | 2* | 0.29* |
| **Domains** | **Attribute Items** | **Median** | **Inter-quartile Range** | **Level of Agreement** |
| **Mindfulness** | 1. I pay attention to how my emotions affect my thoughts and behaviour with talking with patients | 4.00 | 1.00 | 1.00 |
|  | 1. I can easily put my beliefs, opinions, and expectations into words when talking with patients | 3.00 | 0 | 0.92 |
|  | 1. I find it difficult to stay focused on what’s happening in present meetings with patients. (REVERSE) | 2.00* | 1.5* | 0.29* |
|  | 1. I disapprove of myself when I have irrational ideas that appear with patients. (REVERSE) | 2.00* | 1.00 | 0.21* |
|  | 1. When I have distressing thoughts or images during my meeting with a patient, I “step back” and am aware of the thought or image without getting taken over by it. | 3.00 | 1.00 | 0.86 |
|  | 1. I have feelings that I can’t quite identify when talking to a patient (REVERSE) | 2.00* | 1.00 | 0.36* |
|  | 1. Having a daily mindfulness practice is an important part of my work… | 3.00 | 1.00 | 0.79 |
| **Self-Compassion** | 1. When I’m going through a very hard time, I give myself the caring and tenderness I need. | 3.00 | 1.00 | 0.86 |
|  | 1. I’m disapproving and judgmental about my own flaws and inadequacies (REVERSE) | 2.00* | 1.00 | 0.43* |
|  | 1. When I feel inadequate in some way, I try to remind myself these feelings are shared by most people. | 3.50 | 1.00 | 0.79 |
|  | 1. When I’m feeling down, I tend to feel like most other people are probably happier than I am. (REVERSE) | 2.00* | 1.75* | 0.29* |
|  | 1. I give the same attention to myself as I give to patients. | 3.00 | 1.00 | 0.57* |
| **Openness to emotions** | 1. It is easy for me to share my happiness with patients | 3.00 | 1.00 | 0.64* |
|  | 1. Expressing sadness towards colleagues and patients makes me feel anxious/nervous (REVERSE) | 3.00 | 0.75 | 0.79 |
|  | 1. I feel comfortable and reserved when expressing my anger towards a topic brought up by either a patient or a colleague | 3.00 | 1.00 | 0.64* |
|  | 1. I find it difficult to show and describe any of my emotions to colleagues or patients (REVERSE). | 2.5* | 1.00 | 0.31* |
|  | 1. I allow myself to cry with a patient when I feel it is appropriate. | 3.00 | 1.75* | 0.57* |
| **Emotional Intelligence** | 1. I sympathize with the problems that my patients show | 3.00 | 1.00 | 0.86 |
|  | 1. When a colleague or patient annoys me, I stop to think about the other person's situation rather than losing my temper. | 3.5 | 1.75* | 0.71 |
|  | 1. I am able to stay motivated when things do not go well in a meeting when a patient | 3.00 | 1.75* | 0.71 |
|  | 1. Human suffering makes me feel uncomfortable (REVERSE) | 3.00 | 1.5* | 0.71 |
| **Active Listening** | 1. I tend to talk in a in a directive and persuasive way with my colleagues and patients (REVERSE) | 2.5* | 1.75* | 0.5* |
|  | 1. I listen to my patients, paying attention to their unexpressed feelings. | 2.00* | 1.75* | 0.29* |
|  | 1. I’m the kind of person whom patients feel easy to talk to. | 4.00 | 1.00 | 1.00 |
|  | 1. While listening, I get irritated for not understanding the patient’s feelings (REVERSE). | 3.00 | 1.75* | 0.71 |
|  | 1. I struggle to filter out unnecessary information that is not relevant in the conversation (REVERSE). | 2.00* | 2* | 0.36* |
| **Emotional Awareness (Others)** | 1. My patients often say that I have clearly named the emotion that they are feeling now. | 1.5* | 1.75* | 0.29* |
|  | 1. When listening to a patient’s story, I can differentiate between the different emotions that they are talking about. | 3.00 | 1.00 | 0.64* |
|  | 1. My patient’s emotions make sense to me | 3.00 | 0.75 | 0.71 |
|  | 1. I am able to filter out ideas of diagnosis and solutions and instead focus on the patient and what they are experiencing. | 3.00 | 1.00 | 0.78 |
|  | 1. Responding to the patient emotionally is often the most important work done in meetings. | 4.00 | 1.00 | 0.86 |
| **Awareness of Self-Bias** | 1. I can recognize my own biases that could negatively impact a patient | 4.00 | 0.75 | 0.93 |
|  | 1. I know whether my assumptions or ideas could be offensive to a patient or a colleague | 4.00 | 1.00 | 0.86 |
|  | 1. I am aware of my own prejudice and uncertainty | 3.00 | 1.00 | 0.64* |
|  | 1. Self-work is an important part of my professional development. | 3.00 | 1.00 | 0.93 |
|  | 1. Learning to know myself better is an important goal for my professional development. | 4.00 | 1.00 | 1.00 |
| **Self-Disclosure** | 1. I feel confident in opening up and sharing my life experiences to patients and colleagues. | 4.00 | 1.00 | 0.93 |
|  | 1. I am happy to show my whole self as an individual to a network meeting of patients and colleagues. | 3.00 | 1.00 | 0.79 |
|  | 1. I do NOT like to discuss sensitive things about myself to my patient (REVERSE) | 3.00 | 1.5* | 0.71 |
|  | 1. My hobbies and opinions is something that should be hidden from my patients and colleagues (REVERSE) | 2.5* | 1.00 | 0.5* |
|  | 1. I often present a professional side to strangers, as I don’t feel comfortable opening up (REVERSE) | 2.00* | 2* | 0.43* |
|  | 1. I try to share my personal inner thoughts and emotions if I think it may help the patient. | 2.00* | 0.75 | 0.29* |
|  | 1. It is important that I practice sharing my own lived experience. | 4.00 | 1.75* | 0.71 |
| **Knowing when and what to self-disclose** | 1. I know when it is suitable for me to disclose certain attitudes, or experiences of myself towards the patient. | 4.00 | 1.00 | 0.79 |
|  | 1. Self-disclosure can lead to problems if you don’t consider the patients views and situation. | 3.00 | 1.00 | 0.93 |
|  | 1. I understand how powerful words can be. | 3.5 | 1.00 | 0.86 |
|  | 1. I am able to read the mood of a conversation and then decide whether I should say something or not. | 4.00 | 1.00 | 0.86 |
|  | 1. I am aware as to when I may be dominating a conversation and can pull back. | 3.5 | 1.00 | 0.79 |
|  | 1. It is sometimes best to stay quiet than talk. | 3.00 | 1.00 | 0.93 |
|  | 1. It is difficult for me to understand what I can open up with and what I should keep to myself (REVERSE) | 3.00 | 1.00 | 1.00 |
| **Empathy** | 1. An important component of the relationship with my patients is my understanding of the emotional status of the patient and their families. | 2.00* | 1.00 | 0.43* |
|  | 1. I do not allow myself to be touched by intense emotional relationships between my patients and their family members. (REVERSE) | 4.00 | 1.00 | 0.93 |
|  | 1. Attentiveness to my patients’ personal experiences is irrelevant to treatment effectiveness. (REVERSE) | 2.00* | 1.75* | 0.43* |
|  | 1. Because people are different, it is almost impossible for me to see things from my patients’ perspectives. (REVERSE) | 2.00* | 2.75* | 0.43* |
| **Compassion** | 1. I’m quick to notice early signs of distress in patients | 1.50* | 1.75* | 0.30* |
|  | 1. Like me, I know that other patients and colleagues also experience struggles in life. | 3.00 | 1.00 | 0.79 |
|  | 1. When a patient is upset, I try to tune in to how they’re feeling | 4.00 | 1.00 | 0.79 |
|  | 1. When a patient is upset, I try to stay open to their feelings rather than avoid them. | 4.00 | 1.00 | 0.93 |
|  | 1. When I see a patient or a colleague in need, I try to do what’s best for them. | 4.00 | 1.00 | 1.00 |
|  | 1. I consciously consider patients’ feelings, even if they are not expressed. | 3.50 | 2* | 0.64* |
|  | 1. I like to be with patients in their difficulties. | 4.00 | 1.00 | 0.79 |
| **Trust** | 1. It is easy for me to trust patients and colleagues. | 3.00 | 1.75* | 0.72* |
|  | 1. Trusting another person is NOT difficult for me. | 3.00 | 1.00 | 0.64* |
|  | 1. My tendency to trust others is high | 2.50* | 1.00 | 0.50* |
|  | 1. I usually trust people until they give me a reason not to trust them | 2.50* | 11.00 | 0.50* |
| **A Humanistic Approach** | 1. A therapist is a human first, and then they are a human with some expertise. | 3.00 | 0.75 | 0.71 |
|  | 1. Everyone in a meeting are equal, with no right or wrong opinions. | 4.00 | 0.75 | 0.93 |
|  | 1. People need a human to relate and talk to, rather than an ‘expert’. | 4.00 | 1.00 | 0.93 |
|  | 1. Losing the role of an ‘expert’ is hard for me (REVERSE) | 4.00 | 0.75 | 1.00 |
|  | 1. A therapist should have some boundaries between them and the client (REVERSE) | 2.00* | 1.75* | 0.43* |
|  | 1. I am able to go into the therapy as me, rather than as a therapist. | 3.00 | 1.75* | 0.58 |
|  | 1. Being authentic and honest is important skill that I try to practice on a daily basis. | 3.00 | 1.00 | 0.79 |
|  | 1. I am able to care deeply about every patient I work with. | 4.00 | 1.00 | 0.93 |
|  | 1. Just being a fellow human being is sometimes the most important thing we as professionals can offer a person in crisis. | 3.00 | 1.00 | 0.86 |
| **Giving Aware Power (Being Present)** | 1. I always rush to conclusions providing possible answers for the patient (REVERSE) | 4.00 | 0 | 0.93 |
|  | 1. I can put away my experience and knowledge of therapy and enter meetings with a blank state. | 2.00* | 1.75* | 0.30* |
|  | 1. When talking to a patient, over-analyzing is something that I always do and can’t stop (REVERSE). | 3.00 | 1.75* | 0.64* |
|  | 1. I know when it is suitable to provide some of my own experience and understanding and when it is best to listen. | 2.00* | 1.5* | 0.30* |
|  | 1. I am able to listen to my patient, without stepping in and ‘wanting to fix the problem’. | 3.00 | 1.00 | 0.86 |
|  | 1. I feel confident in letting the client lead the conversations/ meetings. | 3.00 | 1.00 | 0.86 |
|  | 1. I am able to filet out ideas of diagnosis, solutions and stay attentive to the patient. | 4.00 | 1.00 | 0.93 |
|  | 1. It is important that I understand how my position of power and privilege influences my professional work. | 3.50 | 1.00 | 0.79 |
| **Accepting** | 1. I view patients for who they are and not based on the diagnosis | 4.00 | 0 | 0.93 |
|  | 1. I am interested more in the person rather than the potential diagnosis that the patient may have. | 3.50 | 1.00 | 0.79 |
|  | 1. I take time to understand the patient and their experiences | 3.50 | 1.00 | 0.86 |
|  | 1. I am good at understanding what someone else’s perspective’s area. | 4.00 | 1.00 | 0.79 |
| **Reflective of One-Self** | 1. I am open to feedback from my colleagues and patients. | 3.00 | 1.00 | 0.79 |
|  | 1. There are always area I can work and improve. | 4.00 | 1.00 | 0.92 |
|  | 1. Being honest to my mistakes is difficult (REVERSE) | 4.00 | 0.75 | 0.93 |
|  | 1. I can reflect on the experiences and feelings that I have experienced with colleagues and clients. | 2.50* | 2* | 0.5* |
|  | 1. When I make mistakes in a meeting, I apologize to the patient. | 4.00 | 0.75 | 1.00 |
| **Tolerating uncertainty and silence** | 1. I can keep an open mind and allow space and time for a patient to reflect. | 3.50 | 1.00 | 0.79 |
|  | 1. Experiencing silence between me and the patient is stressful (REVERSE). | 4.00 | 1.00 | 1.00 |
|  | 1. I will always try to fill in the gaps of silence when talking to patients (REVERSE) | 2.00* | 1.75* | 0.30* |
|  | 1. I can recognize and accept any discomfort present in a meeting between patients and colleagues. | 2.50* | 2.00* | 0.50* |
|  | 1. I get pretty anxious when I’m in a patient meeting that I have no control over. | 3.00 | 1.00 | 0.86 |
|  | 1. When a situation is unclear with a patient, it makes me feel uneasy. (REVERSE) | 2.00* | 1.00 | 0.21* |
|  | 1. I like to plan exactly what I will be doing in a meeting with a patient (REVERSE) | 2.00* | 1.00 | 0.43* |
|  | 1. I like to have things under control when working with patients (REVERSE) | 2.00* | 2.00* | 0.36* |
| **Relationships** | 1. I feel confident that I can form strong meaningful relationships with my colleagues and patients. | 2.50* | 1.00 | 0.50* |
|  | 1. Relationship is an important factor to consider in therapy | 4.00 | 1.00 | 0.86 |
|  | 1. Discussing ideas and feelings together with colleagues and the patient is difficult for me (REVERSE) | 4.00 | 0.75 | 0.93 |
|  | 1. I feel confident in my ability to facilitate more than one voice within a meeting between a patient and their family. | 2.00* | 1.75* | 0.43* |
|  | 1. I place a lot of attention towards the family that surrounds my patient and their relationship. | 4.00 | 1.00 | 0.86 |
| **Meeting Priorities with Patients** | 1. It is important that I understand the patient in order to help them solve their problems. (REVERSE) | 3.00 | 1.00 | 0.93 |
|  | 1. It is important that I am skilled at developing and testing my hypotheses in order to make sure I have understood the patient. (REVERSE) | 2.00* | 1.75* | 0.43* |
|  | 1. My role is often to keep the conversation in the meeting focused on topics that are important for successful treatment. (REVERSE) | 2.00* | 2* | 0.43* |
|  | 1. My primary function is to create space for a dialogue and make sure all voices are heard. | 2.50* | 2.00* | 0.50* |
|  | 1. One of my primary goals is to facilitate an emotional exchange between the patient and their network. | 4.00 | 0 | 1.00 |

Appendix C. Item Statistics for the third-round questionnaire

| Note: * = not meet the criteria of consensus | | | | |
| --- | --- | --- | --- | --- |
| **Domains** | **Attitude Items** | **Median** | **Inter-quartile Range** | **Level of Agreement** |
| **Client-Centeredness** | 1. A thorough diagnosis is unnecessary for effective mental health care. How far do you agree? | 3.00 | 1.00 | 1.00 |
| **General Principles of Mental Health Care** | 1. Mental health care should place emphasis on the client’s words and emotions that are present in the meeting, not the diagnosis prescribed when considering treatment and medication. How far do you agree? | 4.00 | 1.00 | 1.00 |
|  | 1. When working with suicidal or aggressive clients, the priority should be understanding the causes and triggers of the behaviour rather than formulating risk assessments first. How far do you agree? | 3.00 | 0.75 | 0.75* |
| **Peer Support Worker** | 1. Peers (persons with lived experience) provides a different/experiential level of understanding towards a client’s distress, that is important to include in mental health care. How far you agree? | 4.00 | 0 | 1.00 |
| **Trauma** | 1. Most of what is diagnosed as mental illness is the result of trauma. How far you agree? | 3.00 | 0.75 | 1.00 |
| **‘Nothing about them, without them’** | 1. You should not decide on any plans before meeting the clients. How far do you agree? | 4.00 | 0.75 | 1.00 |
| **Personal development** | 1. My personal values and attitudes have a major impact on how I communicate with my clients. How far you agree? | 4.00 | 0.75 | 1.00 |
| **Tolerating Silence** | 1. Tolerating Silence between you and the client has therapeutic benefits. How far do you agree? | 4.00 | 0.75 | 1.00 |
| **Client-Centeredness** | 1. When in a meeting with a client, what is important is your ability to ‘be with them’ rather than ‘doing something to them’. How far do you agree? | 4.00 | 0.75 | 1.00 |

| Note: * = not meet the criteria of consensus | | | | | |
| --- | --- | --- | --- | --- | --- |
| **Domains** | **Attribute Items** | **Median** | | **Inter-quartile Range** | **Level of Agreement** |
| **Mindfulness** | 1. Having a daily mindfulness practice is an important part of my work. How far do you agree? | 4.00 | | 0 | 1.00 |
|  | 1. I am always aware of the feelings that I experience when talking with the client. How far do you agree? | 3.50 | | 1.00 | 1.00 |
| **Self-Compassion** | 1. When I feel down in some way, I try to remind myself these feelings are shared by most people in the service. How far do you agree? | 3.50 | | 1.00 | 0.80* |
|  | 1. I feel comfortable in expressing my sadness and worries in front of colleagues and clients. How far do you agree? | 4.00 | | 1.00 | 1.00 |
| **Emotional Awareness (Others)** | 1. I give less primacy to the ideas of looking for a diagnosis or a solution, and instead, focus on the client and what they are experiencing. How far do you agree? | 4.00 | | 1.00 | 1.00 |
|  | 1. I am aware of my client’s emotions and can acknowledge the feelings that are present in the meeting. How far do you agree? | 4.00 | | 0.75 | 1.00 |
| **Self-Disclosure** | 1. I am comfortable opening up as an individual when talking with clients. How far do you agree? | 4.00 | | 0 | 1.00 |
|  | 1. I am able to discuss sensitive things about myself to the client if it is suitable and safe for both sides. How far do you agree? | 4.00 | | 0.75 | 1.00 |
| **Knowing when and what to self-disclose** | 1. I can disclose certain attitudes, or experiences of myself towards the client if it would be beneficial for both sides. How far do you agree? | 4.00 | | 0 | 0.90 |
|  | 1. I am aware as to when I may be dominating a conversation, giving more control to the client. How far do you agree? | 4.00 | | 1.00 | 1.00 |
| **Compassion** | 1. My client’s emotions are of top priority when considering what medication and treatment are available for their challenges. How far do you agree? | 3.00 | 1.00 | | 0.80* |
|  | 1. Like me, I know that other clients and colleagues also experience difficulties in life. How far do you agree? | 4.00 | 1.00 | | 0.90 |
| **A Humanistic Approach** | 1. A mental health worker is a human first, and then they are a human with some expertise. How far do you agree? | 3.50 | 1.00 | | 0.90 |
|  | 1. Being authentic and honest is an important skill that I try to practice on a daily basis. How far do you agree? | 4.00 | 0 | | 1.00 |
| **Giving Aware Power (Being Present)** | 1. It is important that I understand how my position of power and privilege influences my relationships with clients. How far do you agree? | 4.00 | 1.00 | | 1.00 |
| **Accepting** | 1. I view clients for who they are and not based on the diagnosis. How far do you agree? | 4.00 | 0 | | 1.00 |
|  | 1. I am good at understanding someone else’s perspectives is. How far do you agree? | 3.50 | 1.00 | | 1.00 |
| **Reflective of One-Self** | 1. I am open to feedback from my colleagues and clients. How far do you agree? | 4.00 | 0 | | 1.00 |
| **Tolerating uncertainty and silence** | 1. I can keep an open mind and allow space and time for a client to reflect. How far do you agree? | 4.00 | 0.75 | | 1.00 |
| **Active Listening** | 1. I am able to actively listen to my clients and provide constructive feedback. How far do you agree? | 3.00 | 1.5* | | 0.70* |
| **Self-Reflection** | 1. I am willing to watch myself back on video and reflect on areas that I may need to work on e.g., how I supported dialogue within meetings. How far do you agree? | 3.50 | 1.00 | | 0.90 |

Appendix D. Item Statistics for the four-round questionnaire

| Note: * = not meet the criteria of consensus |
| --- |

| **Domains** | **Attitude Items** | **Median** | **Inter-quartile Range** | | **Level of Agreement** | |
| --- | --- | --- | --- | --- | --- | --- |
| **General Principles of Mental Health Care** | 1. Including an individual’s social network in tackling their mental/emotional issues is an important consideration. How far do you agree? | 4.00 | | 0.00 | | 1.00 |
|  | 1. Providing a rapid response within 24 hours at the point of referral is an important principle to try achieve in mental health care. How far do you agree? | 3.00* | | 0.50 | | 0.75* |
|  | 1. Having the same team members responsible for the care of a client is more effective to their treatment. How far do you agree? | 3.75 | | 0.25 | | 1.00 |
|  | 1. Empowerment of services-users is crucial, mental health treatment should be flexible, for example, clients can decide the number of days and week that are necessary to meet up. How far do you agree? | 3.25* | | 0.25 | | 1.00 |
|  | 1. Most of what is considered symptoms of mental illness, is actually meaningful behaviour. How far do you agree? | 4.00 | | 0.00 | | 1.00 |
|  | 1. The primary goal of mental health treatment should be to increase the agency of the client. How far do you agree? | 3.50 | | 0.50 | | 0.75* |
|  | 1. What you offer and how you help all depends on the needs of the client. How far you agree? | 3.25* | | 1.25* | | 0.75* |
|  | 1. Being open about your feelings and experiences is a necessary skill in mental health treatment. How far you agree? | 3.50 | | 1.00 | | 1.00 |
|  | 1. Mental health care should place emphasis on the client’s words and emotions that are present in the meeting, not the diagnosis prescribed when considering treatment and medication. How far do you agree? | 4.00 | | 0.00 | | 1.00 |
| **Trauma** | 1. Trauma is something that needs to be explored in all client meetings. How far you agree? | 2.75* | | 1.25* | | 0.50* |
|  | 1. The experiences a client has shapes mental health status in later life. How far you agree? | 3.25* | | 1.25* | | 0.75* |
|  | 1. The way mental health services are currently delivered can often be re-traumatizing for clients. How far you agree? | 3.25* | | 1.25* | | 0.75* |
|  | 1. Most of what is diagnosed as mental illness is the result of trauma. How far you agree? | 3.25* | | 1.25* | | 0.75* |
| **Recovery** | 1. Recovering from a mental illness is possible no matter what the situation is. How far you agree? | 2.75* | | 1.25* | | 0.50* |
|  | 1. Clients in recovery sometimes have setbacks. How far you agree? | 3.75 | | 0.25 | | 1.00 |
|  | 1. Clients have different ways in how they recover from mental illnesses. How far you agree? | 3.50 | | 1.00 | | 1.00 |
|  | 1. All people with serious mental illnesses can strive for recovery. How far you agree? | 4.00 | | 0.00 | | 1.00 |
|  | 1. Clients are ‘experts by experience’ who play a role in their own recovery. How far you agree? | 4.00 | | 0.00 | | 1.00 |
| **Client-Centered-ness** | 1. One of the professional’s main function is to try to convey to the client that they are listening and are accepting of the person's feelings and attitudes. How far you agree? | 3.50 | | 0.50 | | 0.75* |
|  | 1. A thorough diagnosis is unnecessary for effective mental health care. How far do you agree? | 3.00* | | 0.50 | | 0.75* |
|  | 1. When in a meeting with a client, what is important is your ability to ‘be with them’ rather than ‘doing something to them’. How far do you agree? | 3.75 | | 0.25 | | 1.00 |
| **Tolerating silence and uncertainty** | 1. Tolerating silence or uncertainty in a client meeting can lead to beneficial outcomes. How far you agree? | 3.50 | | 1.00 | | 1.00 |
|  | 1. If a client wishes to spend time in silence, they should be allowed. How far you agree? | 3.75 | | 0.25 | | 1.00 |
|  | 1. Tolerating Silence between you and the client has therapeutic benefits. How far do you agree? | 3.50 | | 1.00 | | 1.00 |
| **Having no agenda** | 1. Having ‘no fixed objectives’ when meeting clients, allows more free exchange with the client and creates more meaningful experiences. How far you agree? | 3.75 | | 0.25 | | 1.00 |
|  | 1. Rather than focusing on the problem, mental health workers should listen out for meaningful comments and strive to make sense of what the client feels – ‘normalizing discourse’. How far you agree? | 3.25* | | 0.25 | | 1.00 |
| **Peer support worker** | 1. Peer support is an important facilitator of individual mental health recovery. How far you agree? | 3.00* | | 0.50 | | 0.75* |
|  | 1. In mental health teams, Peers (persons with lived experience) are of equal status and value of opinion. How far you agree? | 3.75 | | 0.25 | | 1.00 |
|  | 1. Peers (persons with lived experience) should be involved at every level of service delivery. How far you agree? | 3.75 | | 0.25 | | 1.00 |
|  | 1. Peers (persons with lived experience) provides a different/experiential level of understanding towards a client’s distress, that is important to include in mental health care. How far you agree? | 4.00 | | 0.00 | | 1.00 |
| **Having no ‘expert’ role** | 1. A mental health worker is not there to dominate a client with their ideas but is simply there to create a safe space where the client can talk. How far you agree? | 3.25* | | 1.25* | | 0.75* |
|  | 1. Mental health workers are there to support mutual learning between them and the client, both sides can learn from each other. How far you agree? | 4.00 | | 0.00 | | 1.00 |
|  | 1. Saying less as a mental health worker rather than more is an effective way of treatment care. How far you agree? | 4.00 | | 0.00 | | 1.00 |
| **Family importance** | 1. Understanding a client’s connections in a family is an important step in mental health care. How far you agree? | 3.50 | | 1.00 | | 1.00 |
| **‘Nothing about them, without them’** | 1. Professionals should avoid talking about a client without them being present. How far you agree? | 4.00 | | 0.00 | | 1.00 |
|  | 1. All issues and solutions should be openly discussed with the client for effective therapeutic treatment. How far you agree? | 4.00 | | 0.00 | | 1.00 |
|  | 1. You should not decide on any plans before meeting the clients. How far do you agree? | 4.00 | | 00.00 | | 1.00 |
| **Personal development** | 1. Having a critical understanding my own cultural background helps me provide competent care for persons with cultural backgrounds different from my own. How far you agree? | 3.50 | | 0.50 | | 0.75* |
|  | 1. I need to understand my own life history in order be of help to others. How far you agree? | 3.25* | | 1.25* | | 0.75* |
|  | 1. My personal values and attitudes have a major impact on how I communicate with my clients. How far you agree? | 3.50 | | 1.00 | | 1.00 |
| **Political and social influence** | 1. It is important to consider the political and social factors that may negatively impact a client. How far you agree? | 4.00 | | 0.00 | | 1.00 |

| **Domains** | **Attribute Items** | **Median** | **Inter-quartile Range** | **Level of Agreement** |
| --- | --- | --- | --- | --- |
| **Mindfulness** | 1. I pay attention to how my emotions affect my thoughts and behaviour with talking with clients. How far do you agree? | 3.50 | 0.00 | 1.00 |
|  | 1. I can easily put my beliefs, opinions, and expectations into words when talking with clients. How far do you agree? | 2.75* | 0.50 | 0.50* |
|  | 1. When I have distressing thoughts or images during my meeting with a client, I “step back” and am aware of the thought or image without getting taken over by it. How far do you agree? | 3.00* | 0.50 | 0.50* |
|  | 1. Having a daily mindfulness practice is an important part of my work. How far do you agree? | 2.50* | 0.50 | 0.50* |
|  | 1. I am always aware of the feelings that I experience when talking with the client. How far do you agree? | 3.00* | 0.50 | 0.50* |
| **Self-Compassion** | 1. When I’m going through a very hard time, I give myself the caring and tenderness I need. How far do you agree? | 3.00* | 0.50 | 0.50* |
|  | 1. When I feel down in some way, I try to remind myself these feelings are shared by most people in the service. How far do you agree? | 2.75* | 0.50 | 0.50* |
|  | 1. I feel comfortable expressing my sadness and worries in front of colleagues and clients. How far do you agree? | 3.50 | 0.00 | 1.00 |
| **Emotional Intelligence** | 1. I sympathize with the problems that my clients show. How far do you agree? | 2.75* | 0.50 | 0.50* |
| **Active Listening** | 1. I’m the kind of person whom clients feel easy to talk to. How far do you agree? | 2.75* | 0.50 | 0.50* |
| **Emotional Awareness (Others)** | 1. Responding to the client emotionally is often the most important work done in meetings. How far do you agree? | 2.50* | 0.50 | 0.50* |
|  | 1. I give less primacy to the ideas of looking for a diagnosis or a solution, and instead, focus on the client and what they are experiencing. How far do you agree? | 2.50* | 0.50 | 0.50* |
|  | 1. I am aware of my client’s emotions and can acknowledge the feelings that are present in the meeting. How far do you agree? | 2.50* | 0.50 | 0.50* |
| **Awareness of Self-Bias** | 1. I can recognize my own biases that could negatively impact a client. How far do you agree? | 2.75* | 0.50 | 0.50* |
|  | 1. I know whether my assumptions or ideas could be offensive to a client or a colleague. How far do you agree? | 2.25* | 0.50 | 0.50* |
|  | 1. Self-work is an important part of my professional development. How far do you agree? | 4.00 | 0.00 | 1.00 |
|  | 1. Learning to know myself better is an important goal for my professional development. How far do you agree? | 2.25* | 0.50 | 0.50* |
| **Self-Disclosure** | 1. I feel confident in opening up and sharing my life experiences with clients and colleagues. How far do you agree? | 3.50 | 0.00 | 1.00 |
|  | 1. I am comfortable opening up as an individual when talking with clients. How far do you agree? | 3.00* | 0.50 | 0.50* |
|  | 1. I am able to discuss sensitive things about myself to the client if it is suitable and safe for both sides. How far do you agree? | 3.00* | 0.50 | 0.50* |
| **Knowing when and what to self-disclose** | 1. It is difficult for me to understand what I can open up with and what I should keep to myself (REVERSE). How far do you agree? | 2.25* | 0.75 | 0.25* |
|  | 1. Self-disclosure can lead to problems if you don’t consider the clients views and situation. How far do you agree? | 2.75* | 0.50 | 0.50* |
|  | 1. I understand how powerful words can be. How far do you agree? | 2.75* | 0.50 | 0.50* |
|  | 1. I am able to read the mood of a conversation and then decide whether I should say something or not. How far do you agree? | 2.50* | 0.50 | 0.50* |
|  | 1. It is sometimes best to stay quiet than talk. How far do you agree? | 3.50 | 0.00 | 1.00 |
|  | 1. I can disclose certain attitudes, or experiences of myself towards the client if it would be beneficial for both sides. How far do you agree? | 2.50* | 0.50 | 0.50* |
|  | 1. I am aware as to when I may be dominating a conversation, giving more control to the client. How far do you agree? | 3.00* | 0.50 | 0.50* |
| **Empathy** | 1. I do not allow myself to be touched by intense emotional relationships between my clients and their family members. (REVERSE) How far do you agree? | 1.50* | 0.75 | 0.25* |
| **Compassion** | 1. When a client is upset, I try to stay open to their feelings rather than avoid them. How far do you agree? | 4.00 | 0.50 | 1.00 |
|  | 1. When I see a client or a colleague in need, I try to do what’s best for them. How far do you agree? | 2.75* | 0.50 | 0.50* |
|  | 1. My client’s emotions are of top priority when considering what medication and treatment are available for their challenges. How far do you agree? | 2.50* | 0.50 | 0.50* |
|  | 1. Like me, I know that other clients and colleagues also experience difficulties in life. How far do you agree? | 2.75* | 0.50 | 0.50* |
| **A Humanistic Approach** | 1. Everyone in a meeting are equal, with no right or wrong opinions. How far do you agree? | 3.00* | 0.00 | 1.00 |
|  | 1. People need a human to relate and talk to, rather than an ‘expert’. How far do you agree? | 3.00* | 0.50 | 0.50* |
|  | 1. Losing the role of an ‘expert’ is hard for me (REVERSE). How far do you agree? | 2.00* | 0.70 | 0.25* |
|  | 1. I am able to care deeply about every client I work with. How far do you agree? | 3.25* | 0.00 | 1.00 |
|  | 1. Just being a fellow human being is sometimes the most important thing we as professionals can offer a person in crisis. How far do you agree? | 3.50 | 0.00 | 1.00 |
|  | 1. A mental health worker is a human first, and then they are a human with some expertise. How far do you agree? | 4.00 | 0.00 | 1.00 |
|  | 1. Being authentic and honest is an important skill that I try to practice on a daily basis. How far do you agree? | 4.00 | 0.00 | 1.00 |
| **Giving Aware Power (Being Present)** | 1. I always rush to conclusions providing possible answers for the client (REVERSE). How far do you agree? | 1.50* | 0.75 | 0.25* |
|  | 1. I am able to listen to my client, without stepping in and ‘wanting to fix the problem’. How far do you agree? | 3.75 | 0.00 | 1.00 |
|  | 1. I feel confident in letting the client lead the conversations/ meetings. How far do you agree? | 4.00 | 0.00 | 1.00 |
|  | 1. I am able to filter out ideas of diagnosis, solutions and stay attentive to the client. How far do you agree? | 4.00 | 0.00 | 1.00 |
|  | 1. It is important that I understand how my position of power and privilege influences my relationships with clients. How far do you agree? | 4.00 | 0.00 | 1.00 |
| **Accepting** | 1. I view clients for who they are and not based on the diagnosis. How far do you agree? | 4.00 | 0.00 | 1.00 |
|  | 1. I take time to understand the client and their experiences. How far do you agree? | 3.75 | 0.00 | 1.00 |
|  | 1. I view clients for who they are and not based on the diagnosis. How far do you agree? | 3.75 | 0.00 | 1.00 |
|  | 1. I am good at understanding someone else’s perspectives is. How far do you agree? | 3.75 | 0.00 | 1.00 |
| **Reflective of One-Self** | 1. When I make mistakes in a meeting, I apologize to the client. How far do you agree? | 3.50 | 0.00 | 1.00 |
|  | 1. There are always area I can work and improve. How far do you agree? | 3.50 | 0.00 | 1.00 |
|  | 1. Being honest to my mistakes is difficult (REVERSE). How far do you agree? | 2.00* | 0.75 | 0.25* |
|  | 1. I am open to feedback from my colleagues and clients. How far do you agree? | 3.50 | 0.00 | 1.00 |
| **Tolerating uncertainty and silence** | 1. Experiencing silence between me and the client is stressful (REVERSE). How far do you agree? | 2.25* | 0.75 | 0.25* |
|  | 1. I get pretty anxious when I’m in a client meeting that I have no control over. How far do you agree? | 1.75* | 0.75 | 0.25* |
|  | 1. I can keep an open mind and allow space and time for a client to reflect. How far do you agree? | 4.00 | 0.00 | 1.00 |
| **Relationships** | 1. Relationship is an important factor to consider in mental health care. How far do you agree? | 2.75* | 0.50 | 0.50* |
|  | 1. Discussing ideas and feelings together with colleagues and the client is difficult for me (REVERSE). How far do you agree? | 1.50* | 0.75 | 0.25* |
|  | 1. I place a lot of attention on the family that surrounds my client and their relationship. How far do you agree? | 3.75 | 0.00 | 0.75* |
| **Meeting Priorities with Clients** | 1. It is important that I understand the client in order to help them solve their problems. (REVERSE). How far do you agree? | 2.25* | 0.50 | 0.50* |
|  | 1. One of my primary goals is to facilitate an emotional exchange between the client and their network. How far do you agree? | 3.50 | 0.00 | 1.00 |
| **Self-Reflection** | 1. I am willing to watch myself back on video and reflect on areas that I may need to work on e.g., how I supported dialogue within meetings. How far do you agree? | 3.50 | 0.00 | 1.00 |
